# Obesity and Obesity-Related Disorders—Editorial

**DOI:** 10.3390/ijms25147954

**Published:** 2024-07-21

**Authors:** Grażyna Nowicka

**Affiliations:** Department of Biochemistry and Pharmacogenomics, Centre for Preclinical Research, Medical University of Warsaw, 02-097 Warsaw, Poland; grazyna.nowicka@wum.edu.pl

Obesity and obesity-related complications, including various metabolic diseases and cancers, are significant health problems in developed and developing countries. Although the main causes of excessive accumulation of adipose tissue are known and numerous efforts have been undertaken to fight the obesogenic environment, the number of overweight and obese people continues to grow [1]. As reported by the World Health Organization, since 1990, the worldwide frequency of adult obesity has more than doubled, adolescent obesity has quadrupled, and more than one billion people worldwide are living with obesity [2]. Tackling obesity requires multi-directional and coordinated evidence-based actions involving different sectors [3].

Adipose tissue is an endocrine organ that synthesizes and secretes active molecules regulating physiological functions such as energy metabolism, food intake, and glucose homeostasis [4,5]. There is continuous crosstalk between adipose tissue and other organs [6,7]. Adipose tissue responds to internal and environmental stimuli by remodeling cellular and extracellular components and secretome profiles and expanding through hypertrophy and hyperplasia. This leads to either the preservation of its functionality or the dominance of dysfunctional tissue, which stimulates the development of obesity and related diseases [8,9].

Effective management of obesity and its consequences requires a thorough understanding of the changes in adipose tissue biology under various physiological and pathophysiological conditions affecting body metabolism and health, especially during obesity and weight loss. It is worth noting that adipocyte dysfunction due to epigenetic cell memory, cell senescence, and other phenomena can resist weight loss and persist after weight loss, resulting in a lack of metabolic health improvement and an enhanced risk of weight regain [10]. Additionally, different treatments for obesity may modulate adipose tissue changes in various ways, resulting in more or less favorable effects on metabolic health. Therefore, a deep understanding of adipose tissue biology and its remodeling as both a cause and result of obesity, as well as the molecular links between adipose tissue remodeling, obesity, and other diseases, is necessary for effectively preventing and treating obesity and related disorders.

This Special Issue includes papers on the interaction between lipid droplets and other adipocyte organelles and the influence of different types of molecules synthesized in the human body or arriving from the environment on adipose tissue processes, including cell proliferation and differentiation, new adipocyte formation, lipid accumulation, and thermogenesis. However, the complex and multidirectional effects of the studied factors on adipose tissue biology and limitations in the simultaneous evaluation of the multi-directionality of induced changes and their effects necessitate caution in the proposed translation of experimental study results into clinical practice, as exemplified by the paper by Lai et al. [11] and the related commentary by Cordero et al. [12]. This Special Issue also provides insight into processes relevant to the development and diagnosis of obesity-related diseases, such as non-alcoholic fatty liver disease and atherosclerosis.

It should be emphasized that new drugs effectively reducing body weight [13,14] are now available; therefore, it is particularly relevant to understand their effects on adipose tissue functionality. Research expanding our knowledge in this area is highly anticipated, as it will affect modern obesity management and have the potential for significant long-term improvements in the metabolic health of patients suffering from obesity.

## References

[1] NCD Risk Factor Collaboration (2024). Worldwide trends in underweight and obesity from 1990 to 2022: A pooled analysis of 3663 population-representative studies with 222 million children, adolescents, and adults. Lancet.

[2] GBD 2019 Risk Factor Collaborators (2020). Global Burden of 87 Risk Factors in 204 Countries and Territories, 1990–2019: A systematic analysis for the global burden of disease study 2019. Lancet.

[3] (2023). WHO Acceleration Plan to Stop Obesity.

[4] Chait A., Hartigh L.J. (2020). Adipose Tissue Distribution, Inflammation and Its Metabolic Consequences, Including Diabetes and Cardiovascular Disease. Front. Cardiovasc. Med..

[5] An S.-M., Cho S.-H., Yoon J.C. (2023). Adipose Tissue and Metabolic Health. Diabetes Metab. J..

[6] Pan J., Yin J., Gan L., Xue J. (2023). Two-sided roles of adipose tissue: Rethinking the obesity paradox in various human diseases from a new perspective. Obes Rev..

[7] Wang S., Liu Y., Chen J., He Y., Ma W., Liu X., Sun X. (2023). Effects of multi-organ crosstalk on the physiology and pathology of adipose tissue. Front. Endocrinol..

[8] Iacobini C., Vitale M., Haxhi J., Menini S., Pugliese G. (2024). Impaired Remodelling of White Adipose Tissue in Obesity and Aging: From Defective Adipogenesis to Adipose Organ Dysfunction. Cells.

[9] Xiao Y., Liu D., Cline M.A., Gilbert E.R. (2020). Chronic stress, epigenetics, and adipose tissue metabolism in the obese state. Nutr. Metab..

[10] Della Guardia L., Shin A.C. (2024). Obesity-induced tissue alterations resist weight loss: A mechanistic review. Diabetes Obes. Metab..

[11] Lai H.-C., Chen P.-H., Tang C.-H., Chen L.-W. (2023). Dipeptidyl Peptidase 4 Stimulation Induces Adipogenesis-Related Gene Expression of Adipose Stromal Cells. Int. J. Mol. Sci..

[12] Cordero O.J., Kotrulev M., Gomez-Touriño A. (2024). Comment on Lai et al. Dipeptidyl Peptidase 4 Stimulation Induces Adipogenesis-Related Gene Expression of Adipose Stromal Cells. *Int. J. Mol. Sci.* **2023**, *24*, 16101. Int. J. Mol. Sci..

[13] Melson E., Ashraf U., Papamargaritis D., Davies M.J. (2024). What is the pipeline for future medications for obesity?. Int. J. Obes..

[14] Touceda V., Fontana Estevez F., Cacciagiú L., Finocchietto P., Bustos R., Vidal A., Berg G., Morales C., González G.E., Miksztowicz V. (2023). Liraglutide improves adipose tissue remodeling and mitochondrial dynamics in a visceral obesity model induced by a high-fat diet. Curr. Res. Pharmacol. Drug Discov..

